# High resolution propagation-based lung imaging at clinically relevant X-ray dose levels

**DOI:** 10.1038/s41598-023-30870-y

**Published:** 2023-03-23

**Authors:** Jonas Albers, Willi L. Wagner, Mascha O. Fiedler, Anne Rothermel, Felix Wünnemann, Francesca Di Lillo, Diego Dreossi, Nicola Sodini, Elisa Baratella, Marco Confalonieri, Fulvia Arfelli, Armin Kalenka, Joachim Lotz, Jürgen Biederer, Mark O. Wielpütz, Hans-Ulrich Kauczor, Frauke Alves, Giuliana Tromba, Christian Dullin

**Affiliations:** 1 Department for Diagnostic and Interventional Radiology, University Medical Center Goettingen, Goettingen, Germany; 2 Biological X-ray imaging, European Molecular Biology Laboratory, Hamburg Unit c/o DESY, Hamburg, Germany; 3 Diagnostic and Interventional Radiology, University Hospital Heidelberg, Heidelberg, Germany; 4 Translational Lung Research Center (TLRC), German Center for Lung Research (DZL), University Heidelberg, Heidelberg, Germany; 5 Department of Anaesthesiology, Heidelberg University Hospital, Heidelberg, Germany; 6 Elettra-Sincrotrone Trieste S.C.p.A., Trieste, Italy; 7 Department of Medicine, Surgery and Health Sciences, University of Trieste, Trieste, Italy; 8 Pulmonary Unit, University Hospital of Cattinara, Trieste, Italy; 9 Department of Physics, University of Trieste and INFN, Trieste, Italy; 10 Department of Anaesthesiology and Intensive Care Medicine, District Hospital Bergstrasse, Heppenheim, Germany; 11 Faculty of Medicine, University of Heidelberg, Heidelberg, Germany; 12 Faculty of Medicine, Christian-Albrechts-Universität zu Kiel, Kiel, Germany; 13 Faculty of Medicine, University of Latvia, Riga, Latvia; 14 Department for Haematology and Medical Oncology, University Medical Center Goettingen, Goettingen, Germany; 15 Translational Molecular Imaging, Max-Plank-Institute for Multidisciplinary Sciences, Goettingen, Germany

**Keywords:** Imaging techniques, Translational research

## Abstract

Absorption-based clinical computed tomography (CT) is the current imaging method of choice in the diagnosis of lung diseases. Many pulmonary diseases are affecting microscopic structures of the lung, such as terminal bronchi, alveolar spaces, sublobular blood vessels or the pulmonary interstitial tissue. As spatial resolution in CT is limited by the clinically acceptable applied X-ray dose, a comprehensive diagnosis of conditions such as interstitial lung disease, idiopathic pulmonary fibrosis or the characterization of small pulmonary nodules is limited and may require additional validation by invasive lung biopsies. Propagation-based imaging (PBI) is a phase sensitive X-ray imaging technique capable of reaching high spatial resolutions at relatively low applied radiation dose levels. In this publication, we present technical refinements of PBI for the characterization of different artificial lung pathologies, mimicking clinically relevant patterns in ventilated fresh porcine lungs in a human-scale chest phantom. The combination of a very large propagation distance of 10.7 m and a photon counting detector with $100\,\upmu \hbox {m}$ pixel size enabled high resolution PBI CT with significantly improved dose efficiency, measured by thermoluminescence detectors. Image quality was directly compared with state-of-the-art clinical CT. PBI with increased propagation distance was found to provide improved image quality at the same or even lower X-ray dose levels than clinical CT. By combining PBI with iodine k-edge subtraction imaging we further demonstrate that, the high quality of the calculated iodine concentration maps might be a potential tool for the analysis of lung perfusion in great detail. Our results indicate PBI to be of great value for accurate diagnosis of lung disease in patients as it allows to depict pathological lesions non-invasively at high resolution in 3D. This will especially benefit patients at high risk of complications from invasive lung biopsies such as in the setting of suspected idiopathic pulmonary fibrosis (IPF).

## Introduction

A precise diagnosis and sub-classification of different lung diseases typically requires visualization of pathological changes and structural disease patterns at a high spatial resolution. To date computed tomography (CT) is the primary modality for the imaging of the lung parenchyma by exploiting the intrinsic differences in the x-ray absorption between gas filled alveolar spaces and the pulmonary parenchymal tissue. The individual microstructures of lung parenchyma, such as pulmonary alveoli, interalveolar septa, alveolar ducts or acini lie beyond the spatial resolution capabilities of absorption-based CT, when clinically acceptable radiation dose levels in the range of few mGy are used^1^. In absorption-based CT an increase in resolution demands an enhancement of the applied x-ray dose. If the same level of contrast-to-noise ratio (CNR) should be maintained, the applied dose needs to raised by the power of at least 4^2^. The limited spatial resolution in clinical CT, especially low-dose CT, often results in diagnostic uncertainty and the demand for histological validation which in turn require invasive biopsy procedures^3–5^. Different phase contrast CT imaging techniques, which exploit the refraction of x-rays at structural interfaces within the lung have been proposed and successfully applied to lung specimens and in small animals in-vivo^6–10^. Among them, free propagation phase contrast CT also referred to as propagation-based imaging (PBI) holds the greatest potential for patient applications. PBI is more dose efficient than classical CT and other phase sensitive techniques, since no additional optical elements like phase gratings are needed. Thus, PBI would potentially allow performing tomographic lung imaging at the desired high resolution in patients at relatively low dose levels^11^. However, it has to be noted that PBI requires a sufficient degree of spatial coherence in the incident x-ray beam, which can effectively be achieved only at synchrotron light sources. The principle of PBI^12^ is based on a sufficient sample-to-detector distance allowing the refracted photons to interfere, thereby adding near-field interference patterns to the absorption image. This information can be decoupled by applying single/distance/phase/retrieval algorithms such as the homogeneous form of Transport of Intensity equation (TIE-Hom)^13^. Thus, an additional requirement of PBI is to utilize detectors with sufficiently small detector elements that allow recording of the aforementioned interference patterns, especially since the parallel X-ray beam configuration does not provide an additional magnification factor^14^. However, there is a trade-off between the pixel size of a detector and its dose efficiency. While PBI was shown to be dramatically more dose efficient than classical CT^11^, the CNR decrease due to lower X-ray doses follows a slower kinetic compared to classical CT^15^. Thus, for a potential clinical application a compromise between required resolution, detector pixel size and dose effectiveness must be found. This may explain why relatively few PBI studies apart from the phase contrast breast CT studies at the Italian and Australian synchrotrons^16,17^, have been performed using clinically relevant detectors with pixel sizes of $100\,\upmu \hbox {m}$ and more. Some interstitial lung diseases require spatial resolution beyond the capabilities of clinical high-resolution CT (HRCT) for comprehensive diagnosis. Often only unspecific pulmonary injury patterns such as “ground glass opacity”, “tree-and-bud pattern” or “honey combing” can be detected^18^. For example, the pattern “ground glass opacity” describes partially displaced alveolar air, meaning a lung region with an increased x-ray attenuation, which can be caused by a variety of factors, such as a partial collapse of the air spaces, local thickening of alveolar walls, or liquid filled air spaces^19^. In this study we present an improved set-up for local PBI-based computed tomography providing superior image quality when compared to the state-of-the-art clinical CT, the increase in image quality and spatial resolution using the proposed setup has effectively improved the depiction of simulated pathologies. Additionally, we established a reliable method of dose comparison between the synchrotron setup and the clinical CT using thermoluminescence detectors (TLD’s) to demonstrate dose efficiency of the proposed PBI setup. In the work presented here, we were able to substantially improve our previously published PBI lung imaging method^20^ in terms of image quality as well as by analyzing more realistic pathologies. Thus the presented study presents a further step to realize PBI lung imaging in patients at a dedicated beamline at the Italian synchrotron “Elettra”.

## Results

### The PBI synchrotron setup enables low dose lung imaging on a human-scale

**Figure 1:**
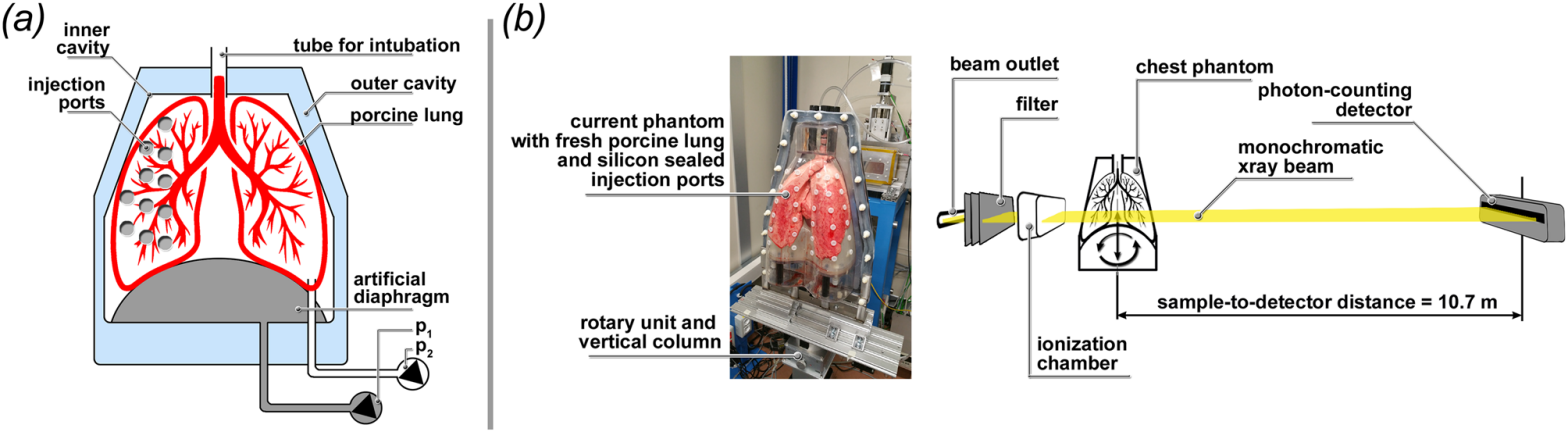
Experimental setup. (**a**) Schematic setup of the human chest phantom. The fresh pig lung is placed in the inner cavity above the artificial diaphragm and attached with the trachea to a tube for external access. One pump ($P_1$) is used to maintain a negative pressure between inner cavity and lung resulting in the inflation of the lung. A second pump ($P_2$) can be used to move the diaphragm, for which different breathing pattern can be programmed. Different outer covers exist that for instance allow to fill the outer cavity with water to mimic the x-ray absorption of the chest. Here we used a single cover with sealed injection ports that allowed injection of artificial tumor nodules in the lung. (b) Shows the phantom mounted up-right on top of the rotary unit in front of the beam outlet and the ionization chamber. The photon counting detector was mounted 10.7 m upstream to provide sufficient sample-to-detector distance to successfully exploit PBI.

**Figure 2:**
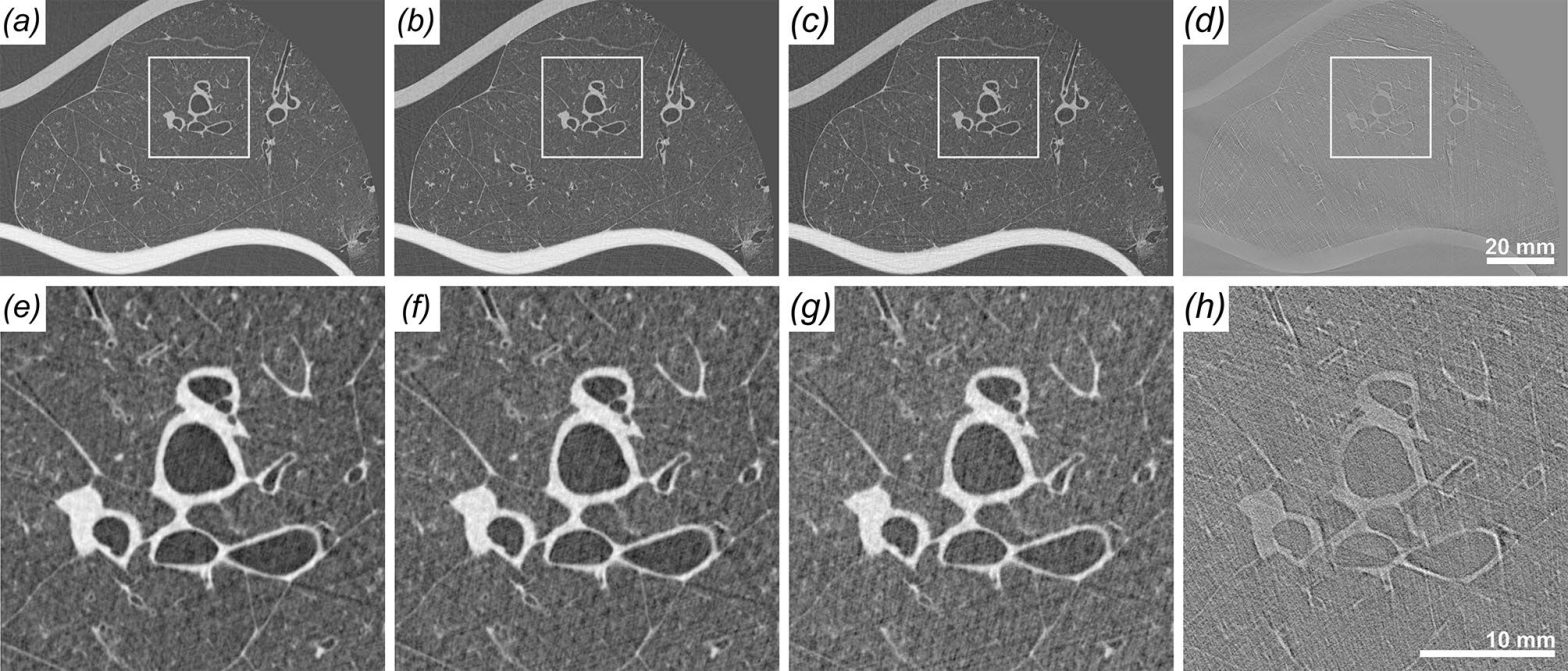
Comparison of the image quality of a healthy pig lung at different dose levels. To simulate lower entrance doses reconstructions with less angular projections are shown. (**a**) Full 8100 projections, entrance dose 10 mGy. (**b**) Half 4050 projections, entrance dose 5  mGy. (**c**) Quarter 2025 projections, entrance dose 2.5 mGy. (**d**) Full 8100 projections, entrance dose 10 mGy, no phase retrieval applied. (**e**), (**f**), (**g**), (**h**) show zoomed in parts of the images above. No visible difference are found between full and half dose while for a quarter of the dose the image quality diminishes a bit. Without phase retrieval the lung structure can barely be identified.

The optimal distance for PBI imaging depends on the size of the features of interest as well as on the pixel size of the detector. Thus, typically only detectors with small pixel sizes are used, which despite the dose efficacy of PBI might still require too much dose for a realistic patient application. Here, to image features of clinical interest we used a detector with “only” $100\,\upmu \hbox {m}$ pixel spacing and a propagation distance of 10.7 m, which in combination with the magnification factor of the slightly cone shaped beam provided an effective pixel size of $67\,\upmu \hbox {m}$. The setup is shown in Fig. 1. To simulate different entrance doses, the acquired data was reconstructed using receding numbers of projections: First, the entire 8100 projections with an entrance dose of 10 mGy (Fig. 2a), second, every $2^{\textrm{nd}}$ projection resulting in an effective dose of 5 mGy (Fig. 2b) and third, every $4^{\textrm{th}}$ projection leading to an effective dose of 2.5 mGy (Fig. 2c). To demonstrate to which degree the image quality is influenced by phase contrast, Fig. 2d,h show the reconstruction of the full 8100 projections without phase retrieval. As shown in the detail views in the second row of Fig. 2 no significant reduction in image quality can be observed between the reconstructions obtained at 10 (Fig. 2a,e), 5 (Fig. 2b,f) and 2.5 mGy (Fig. 2c,g). At all simulated entrance dose levels detailed anatomical substructures such as the secondary pulmonary lobule and the fine intralobular septa can easily be depicted. Without using phase retrieval (Fig. 2d,h), only the main bronchi can be seen, whereas distal substructures of the peripheral lung remain unrecognizable.

### Quantitative comparison of image quality

**Figure 3:**
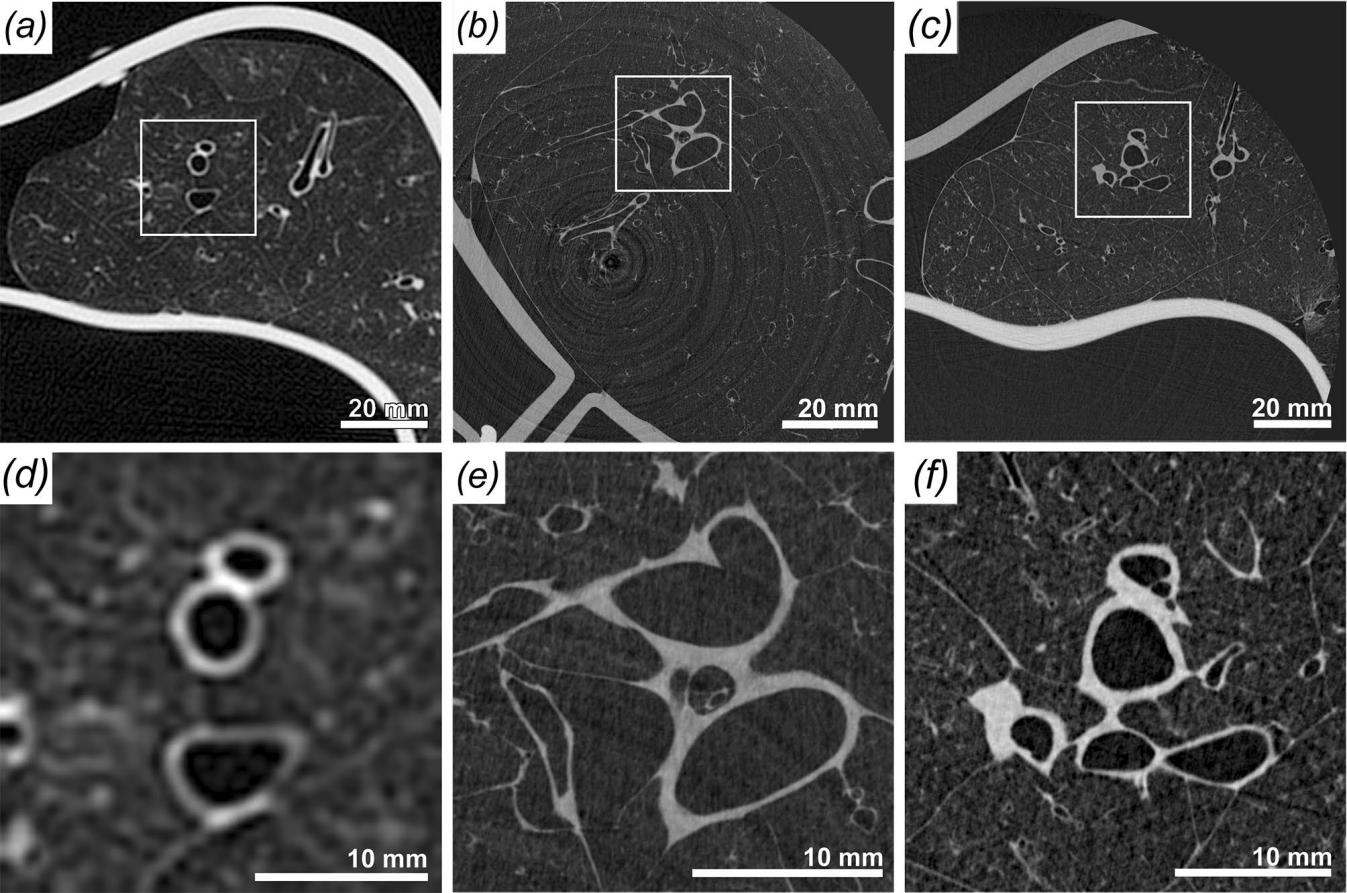
Comparison of the different imaging setups based on scans of three different healthy pig lungs. (**a**) High resolution clinical CT, effective pixel size $455\,\upmu \hbox {m}$, entrance dose 13 mGy. (**b**) SyRMeP synchrotron setup, SDD 2.7 m, effective pixel size $89\,\upmu \hbox {m}$, entrance dose 10 mGy. (**c**) SyRMeP synchrotron setup, SDD 10.7 m, effective pixel size $67\,\upmu \hbox {m}$, entrance dose 10 mGy. (**d**), (**e**), (**f**) show zoomed in parts of the images above. Clearly, the Synchrotron data sets surpass the image quality of the clinical HRCT scan. Moreover, the data acquired at lower dose at 10.7 m are of better image quality than the one scanned at shorter sample-to-detector distance.

**Figure 4:**
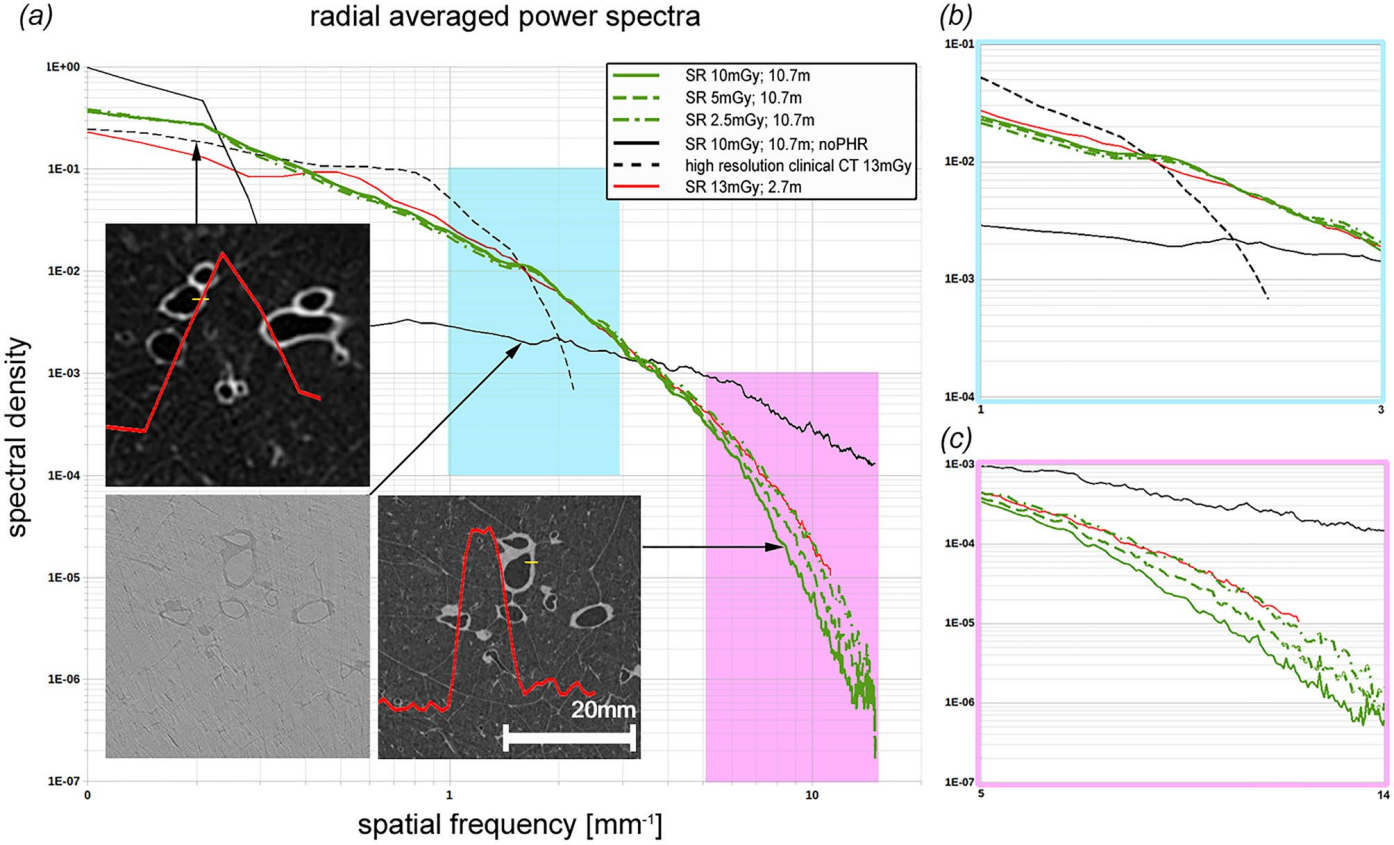
Power spectra. (**a**) Shows radial averaged power spectra of lung regions of interest from the different measurements (clinical HRCT = black dashed, SR with an SDD of 2.7 m = red, SR with an SDD of 10.7 m at 10, 5 and 2.5 mGy green solid, dashed and dot-dashed subsequently the same data at 10 mGy but without phase retrieval is shown in solid black). Clearly, the phase retrieved synchrotron data demonstrates higher powers at medium spatial frequencies and lower powers at high frequencies (noise region) and has therefore a high image quality, which can also be seen in the inserted images. The red curves in the inserts depicts edge profiles at the positions indicated in yellow over a length of 2.5 mm. Despite the high contrast in the clinical data the limited in plane resolution of 450 µm clearly results in edges with a lower sharpness compared to the synchrotron data set. The highlighted region of the medium spatial frequencies (**b**) demonstrates that the images taken at 10.7 m surpass the data taken at 2.7 m at even higher dose of 13 mGy. Moreover, it can be seen that the data at 5 mGy does not deviate much from the results at 10 mGy and that only the data at 2.5 mGy shows a visible drop in power. In the enlarged high frequency domain (**c**) the known effect that phase retrieval acts as a low pass filter and therefore reduces the noise can be seen together with the fact that the noise level depends on the applied dose.

For a comprehensive analysis it is imperative to simultaneously compare features such as contrast, noise, resolution and sharpness of the images. The comparison between synchrotron PBI CT at different entrance doses, propagation distances and the clinical state of the art CT is summarised in Table 1. Here we report the *contrast-to-noise ratio* (CNR) between lung tissue and air and the *edge-enhancement to noise ratio* (EE/N)^21^. EE/N compares the magnitude of the intensity differences at the interface between the background and tissue with the noise in the presumed homogeneous parts of the edge-profiles. In addition, we propose the comparison with an Heaviside function as a model of an ideal edge profile and call this parameter *Deviation from Heaviside function index* (DHI). It should be noted, that reconstruction with a reduced number of projections introduces radial undersampling, which in turn may reduce the effective CNR. At the *sample-to-detector distance* (SDD) of 10.7 m using the complete set of projections with 10 mGy dose a CNR of 18.5 was achieved. At lower entrance doses, simulated by using a lower number of projections for reconstruction, the CNR reduced to 14.9 (5 mGy) and 11.5 (2.5 mGy). In comparison, a shorter SDD of 2.7 m yielded a CNR of 15.4 at a dose of 13 mGy, proving increase efficacy of the PBI at longer propagation distances. Edge quality between long and short propagation distances was preserved with EE/N of 14.1 and DHI of 0.064 (10.7 m SDD) compared to EE/N of 11.78 and DHI of 0.068 (2.7 m SDD). The CNR of 35 in the clinical HRCT acquisition with the dose of 13 mGy is roughly two times higher than the CNR of 18.5 at 10 mGy in the PBI scans at an SDD of 10.7 m. However, the voxel volume of 450 $\upmu \hbox {m}$ x 450 $\upmu \hbox {m}$ x 900 $\upmu \hbox {m}$ is more than 300 times larger than the voxel volume of 67 $\upmu \hbox {m}$ x 67 $\upmu \hbox {m}$ x 67 $\upmu \hbox {m}$ used for PBI. These results are shown in Fig. 3, comparing scans at 10.7 m SDD (a), at 2.7 m SDD (b) and a clinical CT scan (c). Clearly, the clinical data show good contrast, but due to the comparably low resolution it failed to depict the lung structure at the same level of detail shown in Fig. 3d for the PBI scans. Thus, important features of the peripheral lung distal to terminal bronchioles and substructures of the lung’s secondary pulmonary lobule cannot be distinguished from clinical CT images. Since, comprehensive comparison of image quality requires evaluation of the noise level, contrast and edge sharpness simultaneously. To better visualize and perceive the differences in image quality, the parameters are simultaneously displayed in the form of radial average power spectra of clinical data as well as the PBI data at 2.7 m and at 10.7 m SDD with the three different simulated dose levels of 10, 5 and 2.5 mGy in Fig. 4. Spatial frequency describes the structural complexity depicted by the data, with larger structures (bronchi) appearing at the lower frequency, finer structures (alveoli) at higher frequency and noise at the highest frequencies. It can clearly be seen that the middle region of spatial frequencies, which mainly depict the structural content the phase retrieved PBI data (both at 2.7 m = red and at 10.7 m = green) has a larger power than the non-phase retrieved data (black, solid). Additionally, the PBI quality at 10.7 m surpasses that of 2.7 m Fig. 4b. The difference in the power at the very high frequencies, mainly depicting noise can be attributed to the differences in dose. Since the noise level is dose depended, the data acquired at 2.7 m with the highest corresponding dose, shows the least amount of noise Fig. 4c (red). Accordingly, with the decrease in entrance dose, the relative power in the noise region increases (green). Notably with no phase retrieval, the presence of the phase fringes associated with x-ray diffraction contribute to the perceived noise (black).

### Characterization of clinical relevant artificial pathologies

**Figure 5:**
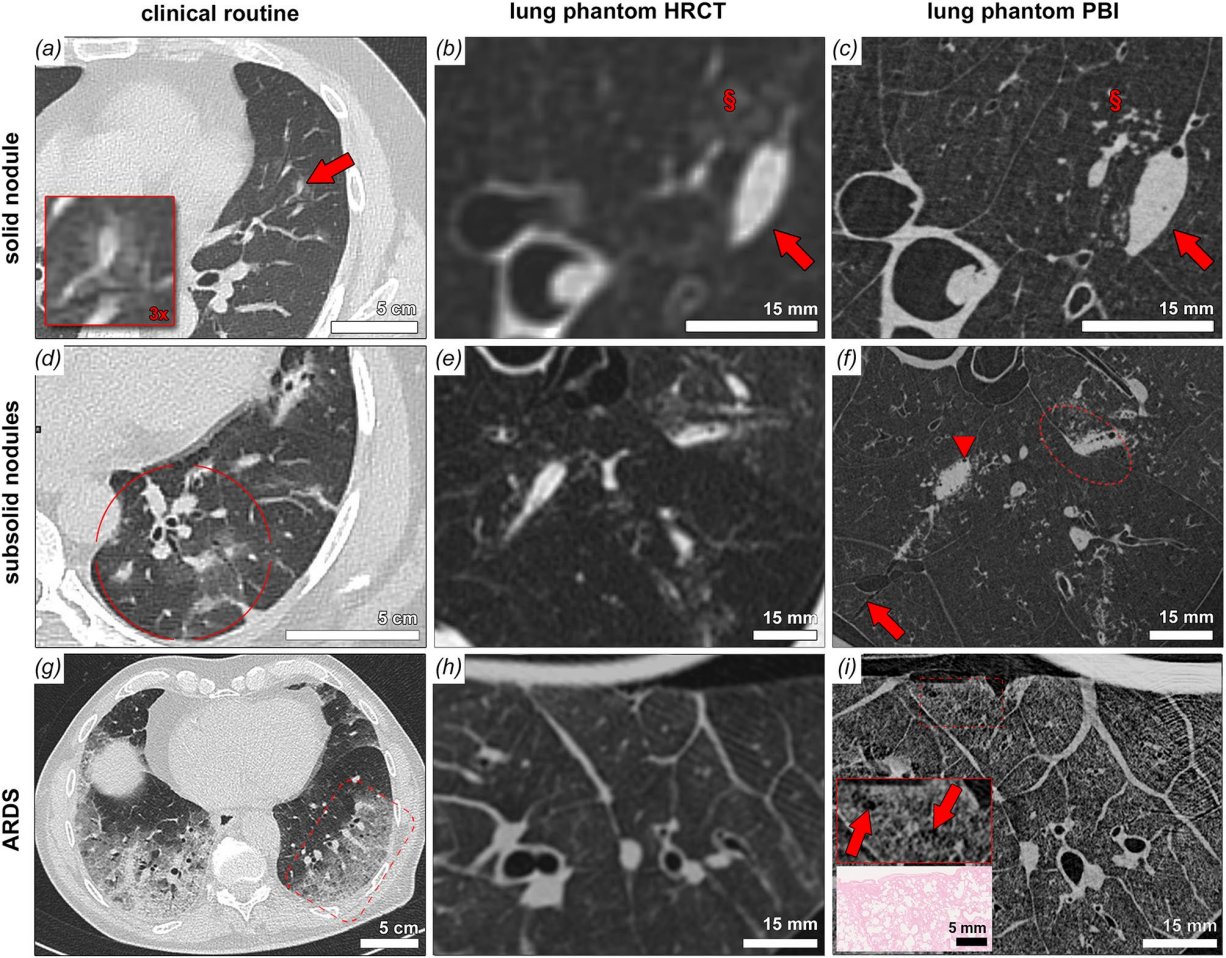
Correlative cHRCT and PBI SRCT with comparative clinical cases. **a**, **d** and **g** show comparative clinical examples of pulmonary damage patterns from cases of viral pneumonia. Patterns of solid nodules (**a**), subsolid nodules (**b**) and acute interstitial pneumonitis/acute respiratory distress syndrome (**g**) are shown. Artificial lesions mimicking those patterns were imaged by clinical HRCT (**b**, **e** and **h**) and the same lung regions were imaged by PBI-based local tomography (**c**, **f** and **i**). For solid nodules PBI-SRCT proofs superior characterization of the lesions borders and substructures (arrow), note the clear depiction of tiny gas enclosures and the orientation of the nodule alongside an interlobular septum. Also a faint ground glass area (§ in **b**) is unveiled as micro tree-in-bud (§ in **c**) by PBI-based SRCT. Grouped subsolid nodules can be depicted in detailed context to the surrounding lung parenchyma. In the circle in (**f**), an extension of the solid component into the interlobular septum can be noted and tiny substructures of the solid component can be depicted (air enclosures indicated by arrowhead in **f**). Perifocal micro tree-in-bud patterns can be attributed to the ground glass components and a parenchymal laceration can be noted (arrow in **f**). In cases of acute alveolar damage (**g**–**i**) substructures of the secondary pulmonary lobule become accessible by PBI-based SRCT, note the heterogeneous distension of individual alveolar air spaces (box in **h**, arrows) with correlative H&E stained histology of the same specimen.

To evaluate the advantages of PBI CT when it comes to identifying and characterizing clinically relevant pathological pulmonary abnormalities, artificial pathologies were induced by agarose injection into fresh healthy porcine lungs and imaged with both clinical HRCT and PBI CT^20^. Figure 5 displays a compilation of clinically relevant pulmonary damage patterns: The first column shows comparative clinical cases, the middle column visualizes artificial lung pathologies as depicted by clinical CT and the third column displays the same finding in PBI CT. Figure 5a–c address solid lung nodules (red arrow), Fig. 5e–f show sub-solid lung nodules (red circle) and Fig. 5g–i depict changes of acute interstitial pneumonitis.

For the imaging of solid pulmonary nodules, clinical imaging data Fig. 5a,b may lack the necessary spatial resolution for a comprehensive characterization of the nodule border and the nodule micro environment. Comparing the Fig. 5b and e with Fig. 5a and d respectively a close resemblance of these structures with the clinical situation can be confirmed. In Fig. 5b a very faint ground glass opacity corona (§) can be noted on clinical HRCT in the periphery of the nodule (red arrow). PBI-CT reveals a micro-tree-in-bud pattern to be responsible for those faint ground glass changes (Fig. 5c). From a clinical diagnostic perspective this information would be of additional diagnostic value, as tree-in-bud phenomena usually represents endobronchial spread of infection. The comparative clinical CT image stems from a case of viral pneumonia.

Figure 5d-f address pattern of multiple sub-solid nodules. The comparative clinical images (Fig. 5d) from a case cryptogenic organizing pneumonia shows nodules with solid components and varying degrees of ground glass portions. The mimicked artificial pattern in HRCT shows similar characteristics (Fig. 5c). The same region displayed by PBI-CT portrays the integration of the various lesions within the hallmarks of peripheral lung anatomy. Individual secondary pulmonary lobules are clearly delimitable and the lesions can be found to be extending within interlobular septa. Ground glass changes in HRCT can be unveiled as micro-tree-in-bud (individual filled alveoli) and a traumatic laceration of lung parenchyma becomes visible due to the increase in spatial resolution and image detail.

The pattern of diffuse parenchymal damage is addressed with a comparative clinical case of acute respiratory distress syndrome (ARDS) (Fig. 5g) and a porcine model of ARDS. Note the superior depiction of sublobular structures such as intralobular septa and perivascular alveolar opacification in PBI-CT (Fig. 5i) compared to clinical HRCT (Fig. 5h). The inserted histological image in Fig. 5h demonstrates that PBI allows to depict the same pattern of heterogenous distension of individual alveolar air spaces, which can not be resolved in the clinical CT data.

### K-edge subtraction imaging

**Figure 6:**
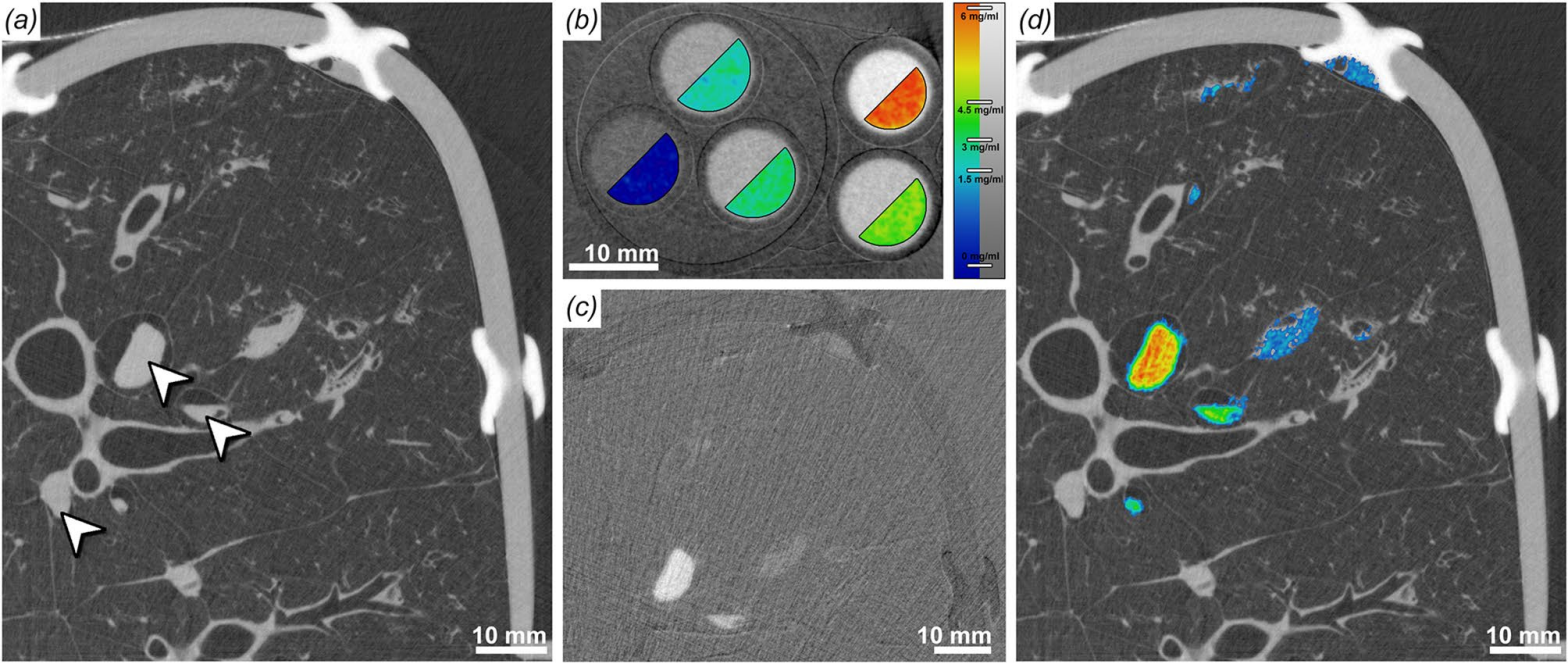
Iodine k-edge subtraction imaging (KES). (**a**) Shows a representative cross-section through a scan (33 keV) of a pig lung injected with different concentrations of iodine/agarose gel mixes. The white arrows point to vessels filled with the contrast agent. Differences in iodine concentration can not be observed. (**b**) CT scan of a phantom with known iodine concentrations (overlaid in pseudo-color), from which an iodine concentration calibration function was retrieved (color bar right). (**c**) The Subtraction image of two scans at 33.5 and 33 keV demonstrates a high specificity for iodine, which in turn allows to quantify difference in the iodine concentration. (**d**) Quantification of the iodine concentrations (color coded) using the calibration function (**b**).

A strong advantage of using quasi-monochromatic X-ray energies provided by a synchroton x-ray source is the possibility to perform K-edge subtraction imaging (KES) as, for instance, described by Bayat et al^6^. The K-edge of an element is the energy at which an electron is emitted from the k shell of the atom which coincides with its absorption maximum. In KES imaging two scans are performed at energies above and below the K-edge. Theses images are then subtracted, resulting in specific contrast for that element. Clinically most relevant in this perspective is the K-edge of iodine as most intravenously applied contrast agents contain iodine. Therefore, we injected agarose gel mixed with different concentrations of iodine. Clearly the highly absorbing iodine can be seen in the filled vessels Fig. 6a (white arrows), but it is indistinguishable from other highly absorbing structures in the tissue like the bronchial wall. We performed two scans at beam energies of 33.0 and 33.5 keV around the iodine K-edge at 33.2 keV. In addition, we imaged a phantom containing known concentrations of the iodine based contrast agent (Accupaque$^{\textrm{TM}}$ 300,GE Healthcare Buchler GmbH and Co.KG) mixed in 1% agarose gel (Fig. 6b). Since the absorption of other materials does not change much in this small energy window, subtracting the images from each other generates a specific iodine map Fig. 6c. Therefore, using the phantom data for calibration, we were able to measure the different iodine concentrations in the specimen as shown in the color coded overlay in Fig. 6d. The central vessel shows an iodine concentration of about 5–5.5 mg/ml, the next smaller vessel a concentration of roughly 4 mg/ml and the other regions of about 1.5 mg/ml (Fig. 6d).

### Pretargeting and lung deformation

**Figure 7:**
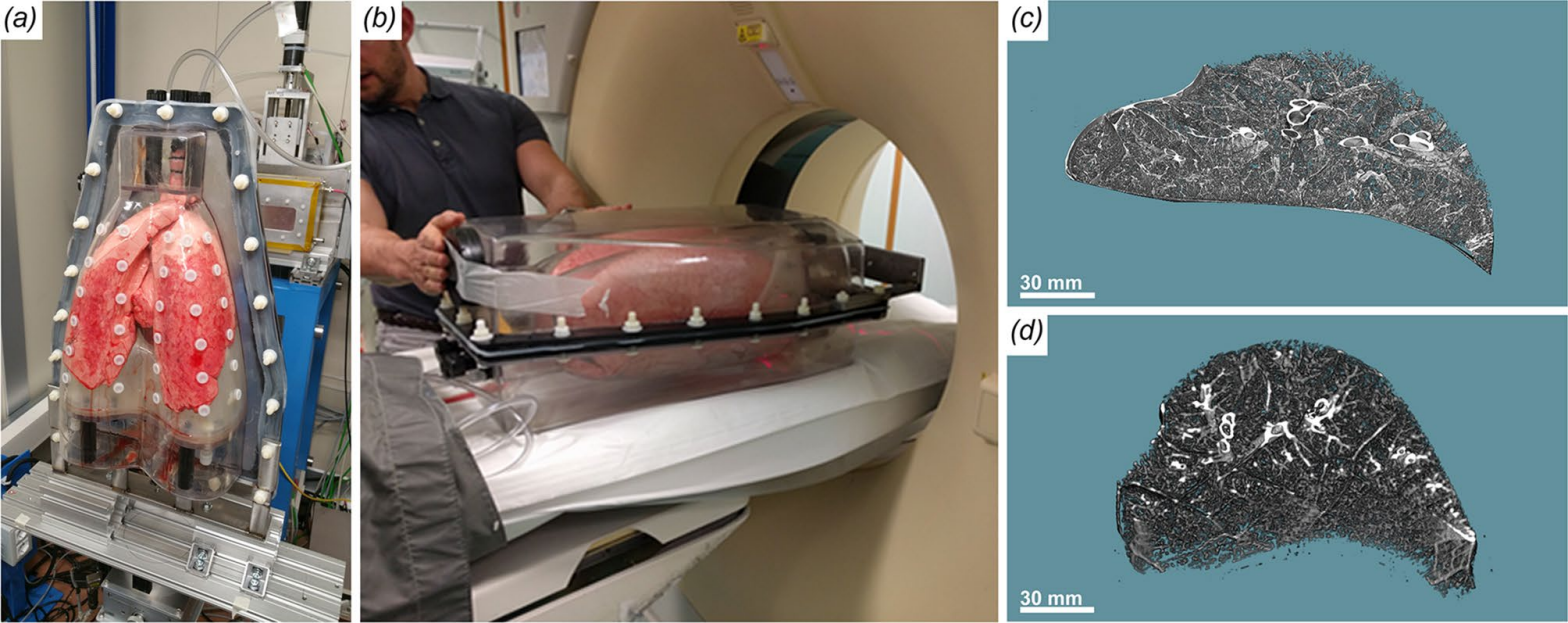
Comparison of synchrotron CT and clinical CT scanning of the human chest phantom. (**a**) Phantom mounted in up-right position at the synchrotron setup. (**b**) Phantom placed in horizontal orientation in the clinical CT scanner. (**c**) Sample lung region extracted from the synchrotron data. (**d**) The same lung region (from the same lung) extracted from the clinical CT. Clearly, the shape of that region differs dramatically between the two setups.

Due to the limited vertical beam size of about 4 mm, even in combination with a potential beam expander, a scenario in which PBI can be applied for lung imaging in patients without a pilot scan in a clinical CT for pretargeting seems unlikely. Thus, we compared the appearance of the same lung region in both modalities. In Fig. 7 the vertical orientation of the phantom at the synchrotron setup (a) and the horizontal position in the clinical CT scanner (b) is shown. We isolated the same area of one lung that was scanned in both modalities without being removed from the phantom. Despite the obvious difference in image quality a strong deformation between the two scans can be observed in Fig. 7c and d. Thus, predefined target regions in the clinical CT can not easily be transferred to the synchrotron scan.

## Discussion

In this study we demonstrate the feasibility of using propagation-based imaging (PBI) at the SYRMEP beamline of the Italian synchrotron “Elettra” for lung imaging in an anthropomorphic human chest phantom. We achieved 7/times higher in plane resolution in combination with even lower x-ray dose levels than in clinical high/resolution lung CT, by utilizing a photon counting detector with a pixel size of $100\,\upmu \hbox {m}$ in combination with a sample-to-detector distance of 10.7 m. For precise dose measurement, thermo-luminescence dosimeters were attached to the phantom. A subset of three lungs was imaged with both the synchrotron imaging setup and a state-of-the-art clinical CT without removing them from the phantom to enable direct comparison of the different imaging modalities. Quantitative comparison of image quality was performed using measures that incorporate contrast, resolution, noise and edge sharpness. Iodine agarose gel mixes were instilled or injected to simulate clinically relevant lung pathologies. In addition, lungs from a porcine acute respiratory distress symptom (ARDS) model were imaged. K-edge subtraction imaging for iodine successfully quantified ascending iodine concentrations in artificial pathologies, showing the greater sensitivity of the synchrotron setup for small changes in the clinical contrast agent concentration.

PBI is a simple X-ray imaging technique, which exploits the wave nature of the X-rays without the need for additional optical elements^12^. PBI-based lung imaging was shown to be dramatically more dose efficient than the classical absorption based clinical CT^11^. Therefore, PBI would not only be suitable for patient lung imaging, but would be extremely beneficial by providing higher resolution images at similar dose levels. However, PBI requires a coherent x-ray source^12^ and a detector with a small pixel size to be utilized successfully^14^. Small detector pixels generally come with the disadvantage of requiring more photons and therefore more dose. Therefore, a fine balance between pixel size, dose and resolution has to be established. Here we used a photon counting detector with a pixel size of $100\,\upmu \hbox {m}$ in combination with a sample-to-detector distance of 10.7 m to perform imaging at dose levels of 2.5–13 mGy, which is even lower than dose levels used clinically in high-resolution CT (HRCT). In comparison to HRCT we achieved 600/times smaller voxel volumes, while CNR and EE/N values were only 50% lower than in clinical CT. The design of the beamline limits the maximum photon energy to 40 keV. Since the contrast in PBI is predominately based on refraction, rather than on absorption, it drops less rapidly with increasing photon energy than absorption contrast^15^. Thus, the x/ray dose for the patient can be potentially decreased even further if higher photon energies are applied. We demonstrated, that a dose as low as 2.5 mGy was reached by reducing the amount of the acquired angular projections, without compromising image quality. This is a result of the extremely low noise level of the photon counting detector and the low pass filtering effect of the applied phase retrieval algorithm. We assume that the required dose could be lowered further by using noise suppression techniques^22^ or iterative reconstruction methods^23^. This would allow to utilise detectors with even smaller pixel sizes, which will increase the image quality even further due to the ability to sample local interference effects even better. It has to be mentioned that using a phantom of up to 45 kg in combination with an imaging method with $100\,\upmu \hbox {m}$ resolution poses strong technical challenges. Due to this, we were only able to realize a rotation speed of $1^{\circ }$ per second, which will be improved in the future to bring the setup to acquisition times compatible with breath hold imaging in patients. Moreover, while the sample translator was able to support the heavy load, the positioning of the specimen in vertical direction was not precise enough to enable stitching of acquisitions at multiple z-positions. In addition, the detector was a prototype which showed some non-linear dose response effects for pixels close to the edges of the internal modules, which resulted in ring artifacts. Interestingly, the low dose acquisitions showed less ring artifacts as the acquired bright field images were able to compensate these effects better. In conclusion, we believe that local PBI lung CT in patients will be technically feasible at $100\,\upmu \hbox {m}$ (or even better) resolution at the same dose levels currently used in clinical whole chest CT. Among other improvements, we primarily aim for acquisition times of less than 10 s to enable imaging in breath-hold also in sick patients, which will require pivotal technical improvements to the sample/patient rotator. Furthermore, additional dose reductions will be addressed.

The use of the monochromatic CT setup at the SYRMEP beamline has further advantages in addition to performing PBI. Monochromatic X-ray imaging prohibits the formation of beam-hardening artifacts^24^, which in a patient application with the presence of ribs and spine in the chest region will be an asset.

Since the energy of the monochromatic setup can be precisely tuned, k-edge subtraction imaging (KES)^6^ can be performed. A similar technique—dual energy CT—is used in clinical settings to derive iodine maps and thereby quantify blood perfusion of organs, such as lungs^25^. However, the two energies used in clinical CT still represent the average energies of polychromatic X-ray spectra. KES can therefore be thought of as an idealized dual energy CT application which could be used for example to specifically detect pulmonary arterial embolisms or address the vascular effects of certain pulmonary diseases^26^. Here we demonstrated that with our setup KES at the k-edge of iodine at 33.2 keV can successfully be performed in a fresh porcine lung on human scale. In clinical contrast enhanced chest CT typically a tube voltage between 80 and 120 kVp is used^27^. Depending on the anode material and filtering the average photon energy is typically between 1/3 and 1/2 of the voltage, thus in the range of 27 and 60 keV. Since, dose deposition increases with lower photon energy and given the fact that one advantage of PBI is its superior efficacy at higher photon energies compared to absorption-based CT, it is difficult to predict if PBI-based iodine KES imaging will be a beneficial application in patients. Alternatively since gold is routinely used as an intravenous contrast agent in preclinical CT imaging^28^ and has a k-edge of 80.7 keV it could be a promising potential candidate for combined KES and PBI applications in patients. Modern detectors even allow to perform KES in a single shot acquisition as demonstrated for instance by Brun et al^29^, circumventing the issue of the additional needed dose for KES.

Our data not only verifies that PBI lung CT can be realized on a patient-scale, but by comparing clinical lung CT scans of patients with PBI images of artificially induced lung pathologies, we show that our setup can greatly improve the diagnostic quality of lung CT. Features that cannot be spatially resolved in clinical CT, and are diagnosed under the general term “ground glass opacity”, can now be characterized in detail and small pulmonary pathologies can be evaluated in the context of detailed sub/lobular lung anatomy.

We are confident that the imaging speed can be increased, however in combination with the small vertical beam size it is unlikely that we can achieve the same total acquisition times of a few seconds used in state/of/the/art clinical CTs. In order, to provide a lateral beam size that allows for a sufficiently large FOV within the human lung, a bending magnet is typically used as the x-ray source due to the larger divergence of the generated beam compared to undulators or wigglers. However, the vertical beam size will still be limited. One option could be to utilize a beam expander^30^. In any case, for an acquisition of the entire lung multiple breath-holds maneuvers would be required. This would not only mean discomfort for the patient, it also bears the risk of patient movement between acquisitions. Thus, we believe a more likely scenario would be to use PBI for a subsequent high/resolution local lung CT “virtual biopsy”, following a clinical CT examination used for pre-targeting. We simulated this by scanning three lungs with both clinical and PBI-based CT. Due to different orientations of the phantom (vertical at the synchrotron, horizontal in the clinical scanner) we found rather strong deviations in the shape of the lung. It can be assumed that in patients the shape of the lungs varies less when shifting between supine and upright position than in the phantom. In addition, maintaining adequate negative pressure in the phantom during the transport between the synchrotron and the clinical CT was a challenge, causing additional movement of the lungs. We believe mathematical modelling could be used to reliably transfer the coordinates identified in clinical CT to scan positions at the synchrotron, should the motion problem persist in patients. Another option would be to perform the clinical pre-targeting scan with an upright CT scanner^31^ which recently have been introduced to the market.

We believe that this two-step approach would be especially relevant in patients with inconclusive findings lung CT findings and invasive lung biopsy is required. The strongly increased spatial resolution of our PBI based approach may, in some cases, especially in patients with a high risk of intervention associated complications, present enough microstructural insight to render an invasive biopsy obsolete. We believe that to further investigate this approach artificial pathologies will not be sufficient. We therefore plan to analyze lungs from porcine pulmonary disease models, such as pulmonary fibrosis.

Another interesting approach would be to realize retrospective gated imaging (RG) in patients. RG is a technique in which more than the needed angular projections are acquired in free breathing - typically by performing multiple rotations^32^. Later on, the movement of the chest is analyzed and the projection data is binned accordingly. Thus, with RG the lung could be reconstructed in multiple phases like shown by Dubsky et al^33^. By comparing these phases local elastic properties could be retrieved^34^, which might improve clinical diagnosis of lung diseases. This type of 4D CT has for instance be established by Fardin et al. in rats^35^. However, RG comes with the disadvantage of elevated x-ray dose levels, hence it needs to be further evaluated if this can be realized in patients within the limits of acceptable X-ray doses. In this study we tried to simulate acquisition by modulating the position of the flexible artificial diaphragm of the lung phantom (data not shown). However, at clinically relevant breathing pattern the lung tissue was laterally moving out of the given FOV. Thus, we need to reevaluate this idea upon the planned beam expansion.

## Conclusion

As demonstrated, the strongly increased spatial resolution of our PBI setup allows for a more comprehensive characterization of lung pathologies such as solid and sub-solid nodules and also features of acute alveolar damage, at comparable radiation doses as clinical CT. Therefore, we believe that especially in patients at high risk of complications from invasive biopsies, e.g. in suspected idiopatic pulmonary fibrosis (IPF), PBI could offer a non-invasive alternative in the sense of a virtual-biopsy to deliver the necessary microstructural insight for a confident diagnostic decision making. We also expect that the completely new level of imaging details in-vivo will majorly improve radiomics approaches^36^ and help to refine or generate imaging biomarkers for different lung diseases. We are aware that the envisioned patient lung imaging beamline at the Italian synchrotron will only benefit a small amount of patients, but we believe that once the diagnostic benefit is clinically established that this will encourage the development of suitable x-ray sources. Moreover, this novel beamline will also further experiments at clinically relevant resolutions and dose levels.

## Methods

### Human chest phantom, porcine lung specimens and artificial lung pathologies

A commercially available lung phantom for imaging porcine heart and lung explants (ARTIChest®, PROdesign GmbH, Heiligkreuzsteinach, Germany) was used, as previously described^37^. The system is constructed of a co-polymer container to simulate a human-scale chest that holds an inflated porcine heart-lung explant by continuous evacuation of the artificial lubricant prepped pleural space with a pressure of −2 to −3 kPa, simulating near human physiological conditions. Here we used fresh porcine lung explants to simulate conditions close to the in-vivo situation. Freshly excised heart-lung explants from 10 mature pigs were analysed paying meticulous attention that the lung surface was intact to prevent air leaks.

The protocol for induction of the acute respiratory distress syndrome (ARDS) in a pig model was approved by the responsible committee for animal research (Regierungspräsidium Karlsruhe, No. 35-9185.81/G-147/17). After induction of anaesthesia the pigs were ventilated with an intensive care ventilator (Carescape R860, GE Healthcare, Madison, USA) using an inspiratory oxygen concentration (FiO2) of 0.4 in a pressure-controlled mode with volume guaranty. Anesthesia was maintained by continuous infusion of 6 mg/kg/h Ketanest S (Pfizer Pharma, Berlin, Germany), 3.6 mg/kg/h midazolam and 10-30 mg/kg/h propofol 2% (Propofol, Fresenius Kabi, Bad Homburg, Germany). There was no use of neuromuscular blockers. Adequacy of the depth of anesthesia was regularly assessed by the absence of spontaneous breathing efforts and lack of muscle tone. Also, a tidal volume of 8 ml/kg body weight (bw), an inspiration/expiration ratio of 1:2 and a PEEP of 5 cmH2O was provided. There was no use of neuromuscular blockers. Adequacy of the depth of anaesthesia was regularly assessed by absence of spontaneous breathing efforts and lack of muscle tone. No recruitment manoeuvres were applied through the study period. During the whole experiment pigs were kept in supine position. Acute lung injury was established by surfactant depletion using 0.9% sodium chloride warmed to body temperature, instilled into the endotracheal tube, and then drained by gravity^38^. Lavage was repeated until a ratio of partial arterial pressure of oxygen to inspired oxygen (P/F ratio) $<\,150$ mmHg. After surfactant wash out an injurious mechanical ventilation was applied (Pressure controlled ventilation: Peak inspiratory airway pressure (PInsp) 35 cmH2O, PEEP 0 cmH2O, RR 12/minute, I:E 1:2 and FiO2 1.0) for 120 minutes. Animals remained in deep anaesthesia for the entire procedure for eight hours and were sacrificed at the end of this period. Post-mortem, one whole lung organ block was explanted for the imaging study.

### Generation of artificial nodules

Subsolid artificial lung nodules with ground glass halos were generated by initial particularly rapid bolus injection of 0.1 ml of 1.5% agarose gel (Agarose I [Molecular Biology Grade] Fisher Scientific, Waltham, USA) prepared with 1.0% of a clinical CT contrast agent containing $300\,\hbox {mg}/\hbox {ml}^{-1}$ iodine (Accupaque 300, GE Healthcare, Little Chalfont, UK) at a temperature of $40\,^{\circ }\hbox {C}$ followed by a slower injection of another 0.1 ml from the same syringe. For this injection technique, rapid handling from the warm water bath to the injection port using 1-ml syringes with Luer-lock screw caps, e.g., BD Plastipak Luer-Lock (Becton Dickinson, Franklin Lakes, USA) proved to be essential, as the rapidly increasing viscosity of the small amount of agarose leads to loosening of the needle cap in syringes with a push-fit connection. In addition, artificial patterns of bronchial mucus plugging and tree-in-bud could be generated by targeted injection of 1% agarose gel (Agarose I [Molecular Biology Grade] Fisher Scientific, Waltham, USA) prepared with 1.0% of a clinical CT contrast agent containing $300\,\hbox {mg}/\hbox {ml}^{-1}$ iodine (Accupaque 300, GE Healthcare, Little Chalfont, UK) into peripheral bronchi and bronchioles. Rapid handling was also necessary for this technique, because the agarose mixture only penetrates to the peripheral bronchioles in a low-viscosity state. Closure of defects of the pleura and lung parenchyma by Allgöwer-Donati surgical backstitch suture using a 3-0 monofilament with FS-1 needle (Ethicon, Somerville, USA), was able to produce artificial peripheral image patterns resembling the appearance of fibrosing interstitial lung disease with bronchiectasis. The injection of the agarose gel was performed into the inflated lung mounted in the phantom using the front cover that includes silicon sealed injection ports as shown in Fig. 1b.

### Synchrotron inline phase contrast CT and single distance phase retrieval

For acquisition of the data the SYRMEP beamline of the “Elettra” synchrotron light source was used^39^. Due to the heavy weight of the phantom of approximately 45 kg a dedicated rotary unit was utilized. The whole setup is shown on Fig. 1. The customized XCounter Flite FX2 photon counting detector (Direct Conversion, Danderyd, Sweden) based on CdTe-CMOS with a pixel size of $100\,\upmu \hbox {m}\,\hbox {x}\,100\,\upmu \hbox {m}$ was used and mounted in a $2^{\textrm{nd}}$ hutch downstream the beamline. With this setup we were able to realize a sample-to-detector distance (SDD) of 10.7 m. Data sets with 8100 angular projection images were acquired over $180^{\circ }$ in 180 s. A monochromatic beam with an X-ray energy of 40 keV in combination with 20.15 mm of aluminium filters were used. Prior 3D reconstruction by using classical filtered back projection (FBP), the TIE-Hom single distance phase retrieval algorithm was applied^13^, both implemented in the SYRMEP Tomo Project software (STP, Version 1.3.2)^40^. To demonstrate the gain in image quality realized with this approach we compared this data with data acquired in the previous experiment using an SDD of 2.6 m^20^. For comparison, three lungs within the ARTIChest phantom were scanned at the Cattinara Public Hospital, Trieste, Italy, with an iCT 256 (Philips GmbH, Hamburg, Germany). Scans with different protocols were acquired. In this publication, we used the high-resolution lung CT scans in axial mode with a voxel size of 0.45 mm x 0.45 mm x 0.9 mm, 200 mAs and 120 kVp for comparison.

### CT image quality measures

In order to evaluate the quality of the obtained images the contrast-to-noise ratio (CNR) was calculated using (1) as introduced in^41^.

1 $$\begin{aligned} CNR=\frac{|S_1-S_2 |}{\sqrt{0.5(\sigma _1^2+\sigma _2^2)}} \end{aligned}$$

with $S_i$ and $\sigma _i$ denoting the average signal (brightness) and the noise level measured as standard deviation of the brightness values in homogeneous regions of two tissue types (1 and 2). Since, CNR is affected by changes in the noise level such as low pass filtering it should always be accompanied with a measure of the sharpness of the image such as the edge enhancement to noise ratio EE/N^21^ (2).

2 $$\begin{aligned} EE/N=\frac{P-L}{\sqrt{\sigma _1^2+\sigma _2^2}} \end{aligned}$$

with *P* and *L* being the highest and lowest values along an edge profile and $\sigma _i$ the standard deviations of the brightness values along the profile in regions apart of the edge for the two adjacent tissues (1 and 2). In phase contrast CT due to the strong edge enhancement the shape of an edge can deviate strongly from a typical staircase-shaped profile. Thus, we introduced a measure calculating the deviation from a Heaviside function (3), which represents an ideally shaped edge (4) and compares a measured edge $\acute{e}(x,x_0)$ at the position $x_0$ with a Heaviside function $H(x,x_0)$. The edge profiles were scaled according to the average grey value of the two adjacent tissues ($S_1$ and $S_2$). To reduce the impact of noise always 20 edge profiles have been averaged. The DHI was measured for a profile length *l* symmetrically around the edge. In this way five edges per each scan have been analysed. For simplicity we assume $S_1 < S_2$ otherwise the direction of the edge was inverted.

3 $$\begin{aligned} \acute{e}(x)= & {} \frac{e(x)-S_1}{S_2-S_1} \end{aligned}$$

4 $$\begin{aligned} H(x,x_0)= & {} \left\{ \begin{array}{lr} 0 &{} x\le x_0\\ 1 &{} x>x_0 \end{array}\right. \end{aligned}$$

5 $$\begin{aligned} DHI(x_0,l)= & {} \frac{1}{l}\sum _{-\frac{l}{2}}^{\frac{l}{2}}{|H(x,x_0 )-\acute{e}(x)|} \end{aligned}$$

Thus, an ideal shaped edge should have a DHI close to 0.

It has been demonstrated in various studies that the application of TIE-Hom can result in smoothing of the images. In order to study this effect, radially averaged power spectra were computed as demonstrated in Albers et al^41^.

**Table 1:** Quantitative comparison of the different imaging techniques and settings.

	dose [mGy]	SDD [m]	eff.ps [$\upmu$m]	CNR	EE/N	DHI
SRCT	10	10.7	67x67x67	18.511 ± 2.575	14.103 ± 4.884	0.064 ± 0.008
SRCT	5	10.7	67x67x67	14.940 ± 1.226	12.123 ± 1.798	0.070 ± 0.004
SRCT	2.5	10.7	67x67x67	11.527 ± 1.537	9.865 ± 0.917	0.079 ± 0.006
SRCT no PHR	10	10.7	67x67x67	1.050 ± 0.141	1.190 ± 0.288	0.533 ± 0.112
SRCT	13	2.7	89x89x89	15.439 ± 3.283	11.780 ± 2.541	0.068 ± 0.008
cHRCT	13		450x450x900	35.221 ± 26.751	26.751 ± 11.529	0.068 ± 0.008

### k-edge subtraction imaging (KES)

To artificially mimic different lung diseases we used 1% agarose gel mixed with an iodine containing clinical contrast agent (Accupaque 300, GE Healthcare Buchler GmbH & Co.KG). Mixes with iodine concentrations of 0, 1.5, 3, 4.5, 6 mg/ml were injected into the lung prior scanning via the injection ports on one dedicated front cover of the phantom. To identify and quantify different iodine concentrations within the lung we realized KES-imaging (k-edge subtraction). Two sequential acquisitions were performed with the same acquisition parameters as described above, but with two photon energies (33 and 33.5 keV respectively) around the k-edge of iodine at 33.2 keV.

### Dose measurement

Since dose measurements between clinical CT scans and the synchrotron setup are difficult to compare due to the different beam energies, we embedded 6 thermoluminescent dosimeters (TLD’s) in the human chest phantom prior to the acquisition. s TLDs (GR-200 A, based on LiF:Mg,Cu,P; Hangzhou Freq-control Electronics Technology Co., Ltd) of size 4.5 mm were used. The TLDs were wrapped in parafilm (Sigma Aldrich) to protect them from moisture. The readings can be found in Table 1.

### Ethical statement

No animal was sacrificed for the particular purpose of this study. One lung was obtained from an approved experiment using an ARDS pig model (Regierungspräsidium Karlsruhe, No. 35-9185.81/G-147/17) after its completion. All other lungs were obtained from a licensed slaughterhouse. The preparations passed the regular veterinarian controls of a licensed slaughterhouse and were treated with the same hygiene precautions as fresh meat. Storage, transport, handling and disposal of faunal by-products were registered at the responsible veterinary office. All methods were carried out according to national law, German law and have been reported in accordance with the ARRIVE guidelines. The pigs were sacrificed with the appropriate euthanasia methods, using a captive bolt pistol in case of the pigs from the slaughterhouse and by injecting 10 to 20 mg potassium chloride while maintaining anesthesia in case of the ARDS model.

### Software and statistics

The data has been acquired using HighPic v9.4 (Hamamatsu Photonics, Germany) in combination with the proprietary detector software. Phase retrieval and 3D reconstruction was performed with STP v1.3.2^40^. Clinical CT data sets were converted using a custom made software in C++ to maintain a 16bit gray scale range. For data analysis and rendering we used ImageJ v1.53c^42^, VGStudioMax v3.1 (VolumeGraphics, Germany) and LibreOffice v7.1.4.2 (The Document Foundation). The radial power spectra was calculated in Python and plotted with Matplotlib. Figures were assembled with Photoshop v13.0.1 (Adobe).

## Data Availability

Due to the large size of the data sets they cannot be stored in an online repository. Thus the data is available from the authors on reasonable request to the corresponding author C. Dullin (christian.dullin@protonmail.com).

## Abbreviations

SRCT: Synchrotron CT
cHRCT: Cinical HRCT
PHR: Single distance phase retrieval
SDD: Sample-to-detector distance
eff.ps: Effective pixel size
CNR: Contrast-to-noise-ratio
EE/N: Edge enhancement to noise ratio
DHI: Deviation from Heaviside function index
